# Novel Role for NFAT3 in ERK-Mediated Regulation of CXCR4

**DOI:** 10.1371/journal.pone.0115249

**Published:** 2014-12-16

**Authors:** Keven Huang, Christine Kiefer, Adeela Kamal

**Affiliations:** 1 Department of Oncology Research, MedImmune, Gaithersburg, Maryland, United States of America; 2 Department of Antibody Discovery and Protein Engineering, MedImmune, Gaithersburg, Maryland, United States of America; National Cancer Institute (INCA), Brazil

## Abstract

The G-protein coupled chemokine (C-X-C motif) receptor CXCR4 is linked to cancer, HIV, and WHIM (Warts, Hypogammaglobulinemia, Infections, and Myelokathexis) syndrome. While CXCR4 is reported to be overexpressed in multiple human cancer types and many hematological cancer cell lines, we have observed poor *in vitro* cell surface expression of CXCR4 in many solid tumor cell lines. We explore further the possible factors and pathways involved in regulating CXCR4 expression. Here, we showed that MEK-ERK signaling pathway and NFAT3 transcriptional factor plays a novel role in regulating CXCR4 expression. When cultured as 3D spheroids, HeyA8 ovarian tumor cells showed a dramatic increase in surface CXCR4 protein levels as well as mRNA transcripts. Furthermore, HeyA8 3D spheroids showed a decrease in phospho-ERK levels when compared to adherent cells. The treatment of adherent HeyA8 cells with an inhibitor of the MEK-ERK pathway, U0126, resulted in a significant increase in surface CXCR4 expression. Additional investigation using the PCR array assay comparing adherent to 3D spheroid showed a wide range of transcription factors being up-regulated, most notably a> 20 fold increase in NFAT3 transcription factor mRNA. Finally, chromatin immunoprecipitation (ChIP) analysis showed that direct binding of NFAT3 on the CXCR4 promoter corresponds to increased CXCR4 expression in HeyA8 ovarian cell line. Taken together, our results suggest that high phospho-ERK levels and NFAT3 expression plays a novel role in regulating CXCR4 expression.

## Introduction

CXCR4 belongs to a large family of G protein-coupled receptors that specifically binds to CXCL12, a chemokine also known as stromal derived factor-1 alpha (SDF-1α). Among various biological processes, CXCR4 plays a critical role in WHIM syndrome, HIV entry, cancer progression and metastasis [1]-[3]. While other GPCR family members are overexpressed in few specific cancers, CXCR4 is overexpressed in more than 23 different types of cancer [4]. Since the CXCR4 receptor is critical in the process of hematopoiesis, development, and vascularization, the deregulation of the CXCR4 signaling pathways may contribute to tumorigenesis [1].

The stimulation of CXCR4 by the ligand SDF-1α leads to activation of various signaling pathways including Janus kinase/Signal Transducer and Activator of Transcription 3 (Jak/STAT3), Nuclear factor kappa-light-chain-enhancer of activated B cells (NFκB), Mitogen-activated protein kinase kinase (MEK1/2), and Extracellular signal regulated kinase (ERK) [5]–[8]. In hematopoietic cells, activation of CXCR4 through the Jak/STAT3 signaling pathways leads to cytoskeletal reorganization and cell migration [9]. In many tumor types, STAT3 is constitutively activated and deregulated STAT3 signaling may contribute to the process of tumorigenesis [10]. More recently, small cell lung carcinoma (SCLC) cells lines and primary SCLC tumors show increased phosphorylation of STAT3, and treatment of SCLC cell lines with SDF-1α further increased STAT3 phosphorylation [7]. Additional investigation showed that upon SDF-1α treatment, JAK2 co-immunoprecipitated with CXCR4 supporting the link between the Jak/STAT3 signaling pathway and CXCR4 [7]. CXCR4 mediated cell migration in a human osteosarcoma cell line involves the MEK1/2, ERK, and NFkb signaling pathways [6]. The activation of CXCR4 upon SDF-1α binding also leads to the dissociation of the trimeric G-proteins into Gα monomer and Gβγ dimer. Downstream signaling events triggered by the Gβγ protein result in an increase in intracellular calcium and various protein kinases [11]. This activates a serine/threonine phosphatase calcineurin which triggers the activation and translocation of various transcriptional factors including Nuclear Factor activated in T-cells (NFAT) [12]. NFAT is a ubiquitous transcriptional factor that transactivates many cytokines including Interleukin-2, 3, 4, 12, inflammatory cytokines, and growth factors [13]–[16]. In human peripheral blood lymphocytes, CXCR4 expression is mediated by calcium and calcineurin activity, thus showing the relationship of CXCR4 regulation and the calcineurin-NFAT pathway [12].

The promoter region of CXCR4 is well characterized and the basal CXCR4 transcription is shown to be controlled mainly by two transcriptional factors, a positive regulating Nuclear Respiratory Factor-1 (NRF-1) and a negative regulating Ying Yang 1 (YY1) [17], [18]. Additionally, CXCR4 expression can be upregulated by calcium and cyclic adenosine monophosphate (cAMP) and by various cytokines including IL-2, IL-4, IL-7, IL-10, IL-15, and TGF-1β [18]–[21]. In contrast, inflammatory cytokines such as TNF-α, INF-γ, and IL-1β all have been shown to suppress CXCR4 expression [22]–[24]. Regulation of CXCR4 expression is important in cell migration, transcription, and cellular trafficking. A better understanding of the signaling pathways and transcriptional factors involved in regulating CXCR4 expression is essential in elucidating the role of CXCR4 in cancer.

Although reports of various cancer types showing high levels of CXCR4 expression, we have experimentally observed that cell lines of various solid tumors exhibit weak cell surface CXCR4 expression *in vitro*. Based on our observations, we hypothesized that certain factors and/or pathways may play a role in regulating CXCR4 expression. Report showing cultured mesenchymal stem cells loses CXCR4 expression but is restored when cultured as 3D spheroids [25] further supports our observed low CXCR4 expression and warrants a 3D spheroid condition to study CXCR4 expression. Under normal culturing conditions, we have observed that high CXCR4 expressing cell lines (Jurkat) have low phospho-ERK levels while Low CXCR4 expressing cell lines (HeyA8) have high phospho-ERK levels. We showed that growing HeyA8 ovarian cell line as 3D spheroids increased not only CXCR4 expression but many transcriptional factors with NFAT3 mRNA being increased by more than 20 fold. Taken together with the phospho-ERK observation and NFAT3 upregulation, the data suggests that ERK signaling pathway and NFAT3 transcriptional factor may play an important role in regulating CXCR4 expression. To our knowledge, we describe here for the first time a novel role for ERK and NFAT3 in regulating CXCR4 expression in HeyA8 ovarian cell line.

## Materials and Methods

### Cell culture and antibodies

Ovarian cancer cell lines HeyA8 (ATCC) and ES-2 (ATCC) were maintained in RPMI medium supplemented with 10% fetal bovine serum, 5 mM L-Glutamine, and 25 mM HEPES buffer. Head and Neck cancer cell line FaDu (ATCC), MCF-7 (ATCC) and MDA-MB-231 (ATCC) breast cancer cell lines were maintained in DMEM supplemented with 10% fetal bovine serum, 5 mM L-Glutamine, and 25 mM HEPES buffer. Cells were maintained at 37°C with 5% CO_2_. Anti-human CXCR4 antibody (clone 44717) was obtained from R&D systems. Western blot analysis with anti-CXCR4 was used at 1:1000 dilutions. Anti-NFAT3 antibody (Cat. # A302-769A) was obtained from Bethyl laboratories. Western blot analysis with anti-NFAT3 was used at 1:1000 dilutions. Anti-actin HRP (Cat. # A3854) conjugated antibody was obtained from Sigma-Aldrich. Western blot analysis with anti-actin HRP was used at 1:50,000 dilutions.

### 3D spheroid generation

Cells cultured as described above were trypsinized with 0.25% trypsin-EDTA cell dissociating buffer (Gibco). To generate one 96 well plate (non-treated costar #3788) with 50,000 cells per spheroid, 4.8 × 10^6^ cells were resuspended in 960 µL of 0.25% MethoCult solution (Stemcell). Cells were carefully mixed without producing bubbles by gently pipetting up and down, and 10 µL of the cells/MethoCult mixture were transferred to each well. The 96 well plates were centrifuged at 1500 RPM for 10 minutes to pellet the cells. The pelleted cells/MethoCult mixture was incubated overnight at 37°C with 5% CO_2_. The following day, 200 µL of fresh complete media was added to each well and the spheroids were incubated for an additional 7–10 days prior to analysis. When treating 3D spheroids with NFAT inhibitor cyclosporine A, the drug was added to the fresh complete media on day 2 for an additional 7–10 days until flow cytometry analysis.

### Western blot analysis

Proteins were extracted from human cell lines with RIPA (Radioimmunoprecipitation assay) buffer (150 mM NaCl, 50 mM Tris, 1% NP-40, 0.5% Sodium deoxycholate, 0.1% SDS, 5 mM EDTA) plus protease and phosphatase inhibitor (Sigma) for 30 minutes and spun down at 12,000 RPM at 4°C. For human xenograft tumors, proteins were harvest by processing the tumor using FastPrep-24 tissue homogenizer (MP Biomedical) in 1 mL of RIPA buffer. Protein quantitation was performed using Pierce's BCA assay kit according to manufacturer's protocol. Ten µg of protein was loaded using NuPAGE Novex 4–20% Bis-Tris mini gel from Invitrogen. Gels were run at 150 volts and transferred using the iBlot from Invitrogen. Pierce's supersignal west pico enhanced chemiluminescent substrate was used to detect protein signals.

### Flow cytometry analysis

Cells lines were harvested using Accutase cell dissociating buffer (Gibco) and washed 2X with wash buffer (Dulbecco PBS + 0.5% BSA + 5 mM EDTA). Primary antibody at a concentration of 10 µg/mL was added to 5×10^5^ – 1×10^6^ cells and incubated at 4°C for 2 hours. After primary antibody incubation, cells were washed twice with wash buffer and goat anti-human FITC or APC IgG secondary antibody (Jackson ImmunoLab) was added at a 1:200 dilution along with 7AAD or DAPI for viability staining. After secondary antibody incubation of 1 hour, cells were washed 2X with wash buffer and resuspended in 200 µL of PBS + 2% FBS for flow cytometry analysis. Flow results were occasionally plotted as a bar graph using integrated median fluorescence intensity (iMFI) scale. The formula for iMFI is (iMFI  =  median fluorescence x % positive cells). The integrated approach for graphing the results compared to controls aims to increase the quantitative contents of the data [26].

### Phospho-ERK assay

Cells were grown as described in materials and methods. For the analysis of ERK 1/2 phosphorylation, 100,000 cells were grown in serum free media overnight. Cells were grown in serum free media to prevent potential growth factors in serum that may potentially activate and/or inhibit the ERK pathway thereby reducing assay background. Cells were stimulated the following day in fresh serum free media containing 10 ng/mL of SDF-1α (Peprotech). Cells were harvested with MSD lysis buffer (Meso Scale Discovery). The MSD phospho-ERK/total-ERK detection assay was followed according to manufacturer's protocol (Meso Scale Discovery).

### Real-time PCR and PCR array analysis

RNA was extracted, and total RNA (5 µg) was reverse transcribed using commercially available kits (Qiagen, SABiosciences). Taqman gene expression assays for CXCR4 (Hs00976734_m1), NFAT3 (Hs00190037_m1), and GAPDH control (Hs99999905_m1) were obtained from Applied Biosystems. PCR transcriptional factor arrays were purchased from SABiosciences and performed according to manufacturer's protocol. For the PCR array, approximately 25 ng of cDNA were used for each gene expression assay. Each PCR cycle was performed at 30 seconds at 94°C, 30 seconds at 60°C, and 1 min at 68°C for 40 cycles.

### Drug treatments

ERK inhibitor U0126 (Cell signaling), PI3K inhibitor LY294002 (Cell signaling), FK506 (LC Laboratories), Ionomycin (Sigma Aldrich), and Cyclosporin A (CsA, Sigma Aldrich) were used at the indicated concentrations. Cell treatments of adherent cells with U0126, LY294002, FK506, and Ionomycin were incubated for 48–72 hours. Treatment of 3D spheroids with CsA was incubated for 7 days. Phorbol myristate ester (PMA, Sigma Aldrich) was used at a concentration of 100 ng/mL for 30 minutes prior to SDF-1α treatment.

### Knockdown studies with shRNA and siRNA

Lentiviral vectors for inducible shRNA expression targeting CXCR4 were purchased from Open Biosystems (pTRIPZ). The pTRIPZ vector contains Tetracycline promoter for shRNA induction and puromycin for selection. HeyA8 cells were transduced with lentivirus and puromycin selected for 2 weeks. Tetracycline concentration for shRNA induction was used at 1 µg/mL. NFAT3 siRNA was purchased from Dharmacon (ON-TARGETplus human NFAT3). Transfection of siRNA was performed with lipofectamine RNAiMAX. The ratio of siRNA to lipofectamine was used at a ratio of 1:2.5. Mixture of 10 nM of siRNA and lipofectamine were incubated for ½ hours and transferred to a 6 well plate. For each well, 200,000 HeyA8 cells were plated and incubated for 72 hours prior to protein extraction.

### Transfections and reporter assays

HeyA8 cells were grown under normal culturing conditions. Fifty thousand cells were plated the day before transfections in a 24 well plate. Transfection of Cignal NFAT-Luc reporter (SABiosciences) was performed with Lipofectamine 3000 (Life Technologies). The amount of Lipofectamine 3000 used was 1µl per 500 ng of NFAT-Luc DNA. The mixture of lipofectamine and DNA were incubated for 5 minutes and then added to the cells. Reporter assay was performed according to promega's dual luciferase assays. Cells were lysed in 50–100 µl of passive lysis buffer at room temperature for 15 minutes and 10 µl of samples were transferred to a white walled clear bottom 96 well plates in duplicate. The Wallac 1420 luminometer was used to detect both the luciferase and renilla (normalized internal control) fluorescence. The NFAT relative luciferase unit (RLU) was plotted based on the NFAT luciferase reading divided by Renilla luciferase reading. For the 3D reporter assay, 24 hours after transfecting NFAT-Luc DNA, 3D spheroids were formed and incubated for an additional 6 days for a total of 7 days.

### Chromatin immunoprecipitation

Chromatin immunoprecipitation was performed essentially as described [27]. Briefly, HeyA8 cells grown under adherent and spheroid culture conditions and adherent HeyA8 cells treated with 20µM U0126 were cross-linked in 1% formaldehyde. Chromatin was sheared to an average fragment size of 150–200 bp using both MNase digestion and sonication, and immunoprecipitation was performed using antibodies to NFAT (Bethyl), NFκB (Abcam), and Polymerase II (Santa Cruz) with appropriate isotype controls. Quantitative PCR was performed on two independent immunoprecipitations in duplicate using iQ SYBR Green Supermix (Bio-Rad). Fold changes in occupancy of NFAT, NFκB, and Pol II at the CXCR4 promoter were determined using the comparative Ct method.

### Xenograft Tumors

Human Breast cell lines MCF7 and MDA-MB-231 were maintained as adherent cultures at 37°C in a 5% CO_2_ incubator in OPTI-MEM (MCF7), containing 5% fetal bovine serum (FBS) and Roswell Park Memorial Institute Medium (MDA-MB-231), containing 10% fetal bovine serum (FBS). On the day of implantation, all cells were spun down, and resuspended to a final concentration of 2.5 × 10^7^ cells/mL in a 1:1 mixture of PBS plus Matrigel (MCF7 and MDA-MB-231). Female athymic nude mice were each injected orthotopically in the right mammary fat pad with 5 × 10^6^ cells in a 200 µL volume (MCF7 and MDA-MB-231). The tumors were harvested when the volume size reached > 1000 mm^3^. Tumor volume is calculated using the formula: Tumor volume  =  [length (mm) × width)^2^]/2.

## Results

### HeyA8 ovarian cancer cells express low levels of surface CXCR4

**Figure 1:**
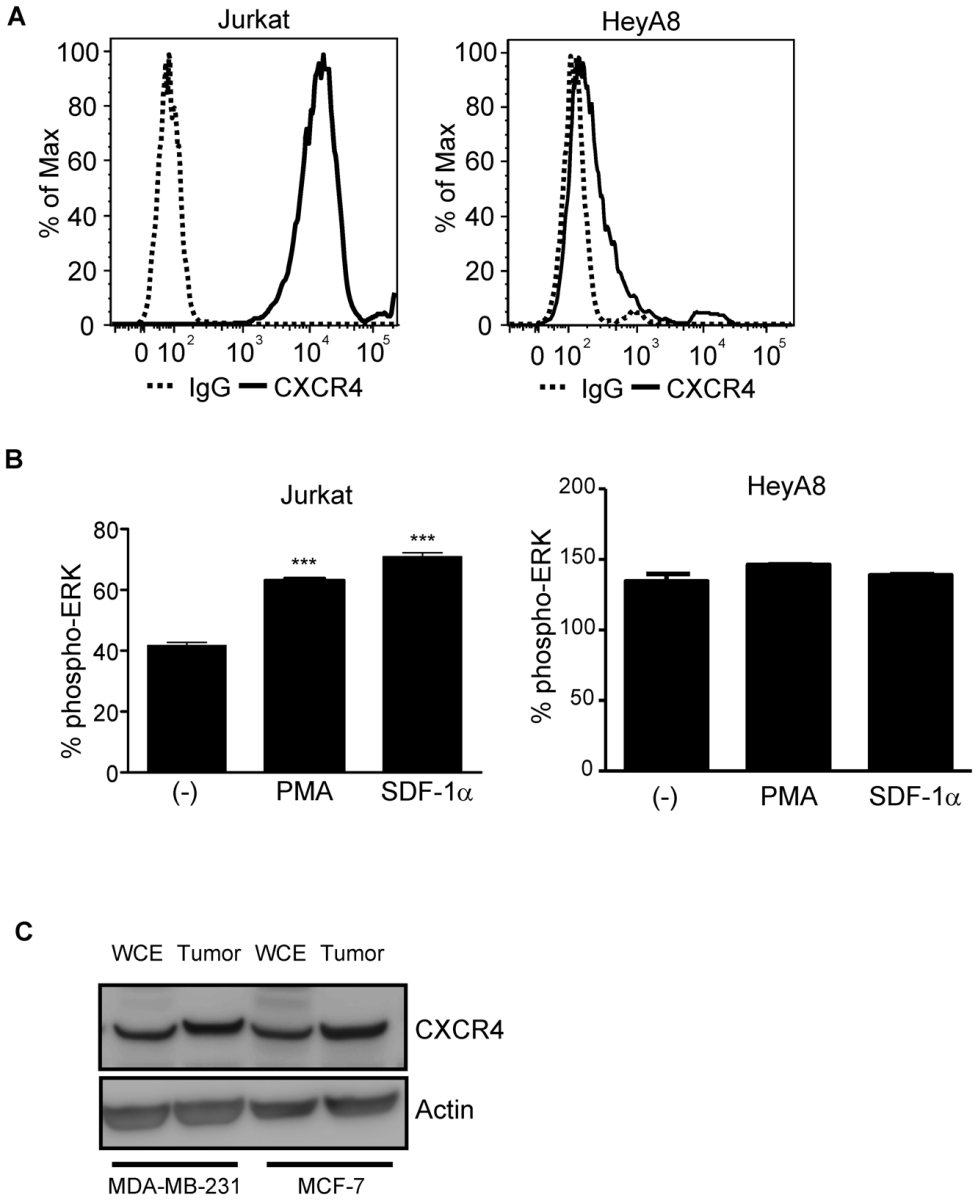
HeyA8 Ovarian cell line expresses low levels of cell surface CXCR4. Jurkat and HeyA8 cells were cultured under normal growth conditions. For flow cytometry analysis, 250,000 cells per conditions were used. For phospho-ERK assay, 100,000 cells per conditions were used. (**A**) Jurkat cells showed high expression levels of surface CXCR4 (solid line) when compared to control IgG (dotted line) whereas HeyA8 ovarian cell line showed low surface CXCR4 expression. (**B**) ELISA based phospho-ERK levels when normalized to total ERK showed increased % phospho-ERK levels in Jurkat cells when stimulated with either PMA (250 ng/mL) or SDF-1α (10 ng/mL). Phospho-ERK levels when normalized to total ERK showed no change in pERK levels in HeyA8 cells when stimulated with either PMA (250 ng/mL) or SDF-1α (10 ng/mL). (***, P <0.05 vs (-), 1-way ANOVA). Each bar graph is representative of at least 2 experiments. (**C**) 10 µg of protein was used for western blot analysis of breast cancer cell line MDA-MB-231 and MCF-7 where whole cell lysates and tumor extracts showed detectable CXCR4 protein levels.

CXCR4 surface expression levels are high in many cell lines such as Molt4 and Jurkat T-cells, IM9 B cells, and MonoMac1 monocytic cells [8]. In agreement with previously published results, we showed that Jurkat T-cells expressed high cell surface expression of CXCR4 (Fig. 1A, Jurkat). To assess CXCR4 expression in solid tumor cell lines, we performed flow cytometry analysis in HeyA8 ovarian cancer cells and showed weak cell surface CXCR4 expression (Fig. 1A, HeyA8). The weak surface CXCR4 expression was surprising and prompted a further evaluation of CXCR4 protein expression. Since SDF-1α mediated activation of NF-kb pathway has been reported to be dependent on the MEK/ERK pathway [8], [28], we set out to determine if HeyA8 adherent cells were responsive to SDF-1α. In contrast to Jurkat T-cells which were responsive to SDF-1α treatment in a phospho-ERK assay (Fig. 1B, Jurkat), the addition of SDF-1α to HeyA8 cells failed to induce phospho-ERK activation which confirmed the low surface CXCR4 expression (Fig. 1B, HeyA8). Upon further comparison between Jurkat and HeyA8 cells, the basal phospho-ERK levels were much higher in HeyA8 cells (Fig. 1B, (−) Jurkat (∼40%) vs. HeyA8 (∼125%)). Western blot analysis of low CXCR4 expressing breast cancer cell *lines in vitro* MDA-MB-231 and MCF-7 of whole cell extracts and tumor xenograft showed CXCR4 protein expression with tumor xenograft having higher CXCR4 expression (Fig. 1C). Using additional adherent cell lines with low CXCR4 surface expression, we investigated whether 3D culturing could alter the levels of CXCR4 expression.

### Effect of 3D spheroids on CXCR4 surface expression

**Figure 2:**
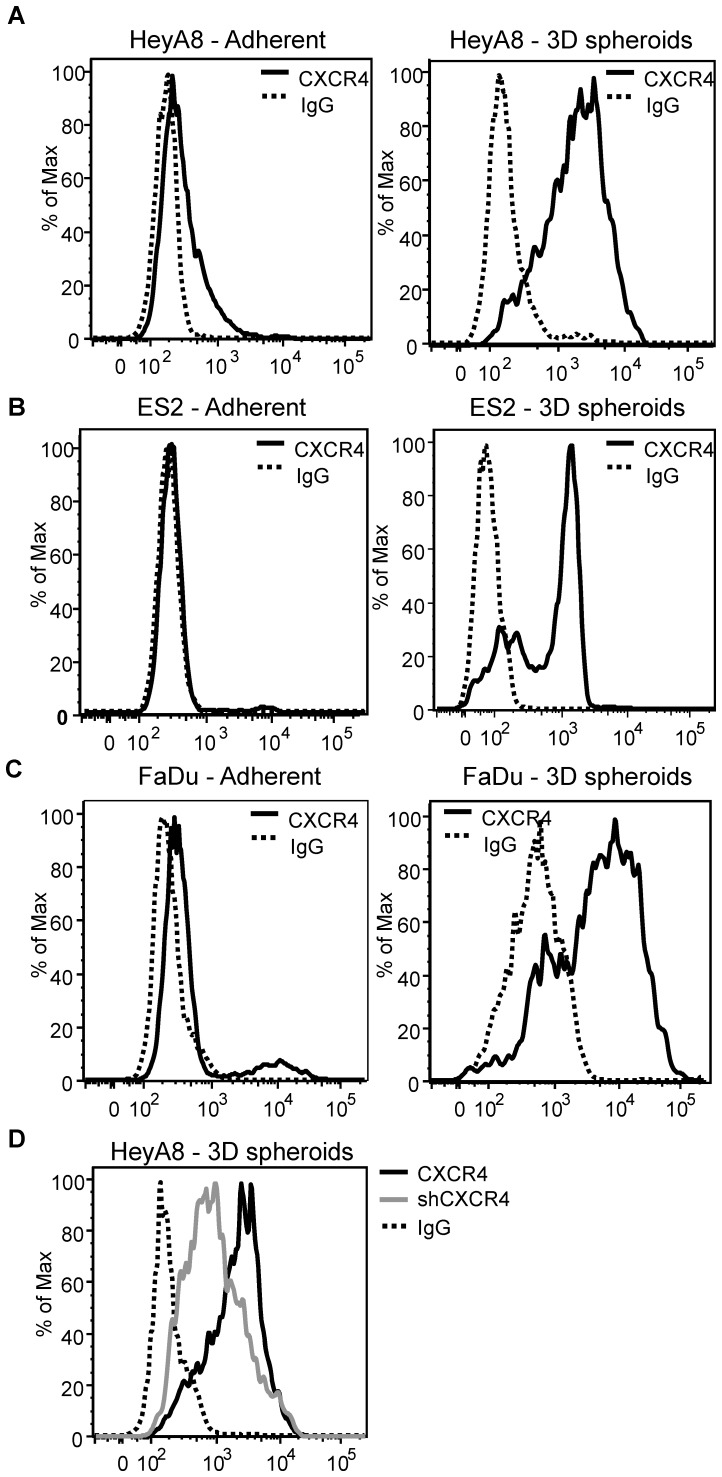
3D spheroid culture condition enhances CXCR4 surface expression. Cell lines were cultured as 3D spheroids as described in materials and methods and dissociated using accutase cell dissociating buffer for 10 minutes for flow cytometry analysis. 250,000 cells were used for the flow cytometry analysis. (**A**) HeyA8, (**B**) ES-2, and (**C**) FaDu cells showed low levels of detectable surface CXCR4 when compared to control human IgG under normal adherent culturing conditions. When grown under 3D spheroids, all of the cell lines showed a dramatic shift in CXCR4 surface expression. (**D**) Tet-inducible shRNA knockdown of CXCR4 (shCXCR4) in HeyA8 cells under 3D spheroid condition showed decreased CXCR4 surface expression (Grey line) indicating the specificity of increased CXCR4 surface expression under 3D spheroids. Flow cytometry of CXCR4 expressions in 3D spheroids were pooled representative cells isolated from at least 10 individual spheroids. Each flow graph is representative of 3 (ES2 and FaDu) or 4 (HeyA8) separate experiments.

Although many hematological tumor cell lines have been reported to express high levels of surface CXCR4, our flow cytometry analysis showed that multiple adherent solid tumor cell lines expressed low levels of surface CXCR4 (Fig. 2A–C, adherent). The discrepancy in CXCR4 expression between adherent solid tumor and suspension cell lines suggests that even though HeyA8 cells express CXCR4, some unknown factors possibly related to growth conditions or pathway deregulation, may alter CXCR4 surface expression. It has been previously shown that cultured human mesenchymal stem cells (hMSC) express low CXCR4 expression but upon culturing hMSC in 3D aggregates, CXCR4 expression is restored [29]. To test the theory of adherent conditions altering CXCR4 expression, HeyA8 ovarian cancer cells were cultured as 3D spheroid and surface CXCR4 expression was determined. HeyA8 adherent cells showed consistent low surface CXCR4 expression, but when HeyA8 cells were cultured as 3D spheroids the levels of surface CXCR4 expression increased significantly (Fig. 2A). To further test whether this phenomenon occurred in other cell lines, ES2 ovarian and FaDu head and neck cancer cell lines were both cultured as 3D spheroids. In agreement with HeyA8 cells, ES2 and FaDu cell lines also showed a dramatic increase in surface CXCR4 expression when cultured as 3D spheroids (Fig. 2B and 2C). Knockdown of CXCR4 using shRNA showed a decrease in surface CXCR4 levels, thus demonstrating the specificity of the detected surface CXCR4 levels in 3D spheroids (Fig. 2D). Taken together, these results suggest the 3D spheroid is a good method to study the regulation of CXCR4 expression. During our investigations into the differences in CXCR4 expression between adherent and suspension cells, we have observed that the phospho-ERK basal level in adherent HeyA8 cells was at a higher level than Jurkat cells (Fig. 1B (-), HeyA8 ∼125%, Jurkat ∼40%). We next investigated the role of phospho-ERK in CXCR4 expression.

### Phospho-ERK level decreases in 3D spheroids

**Figure 3:**
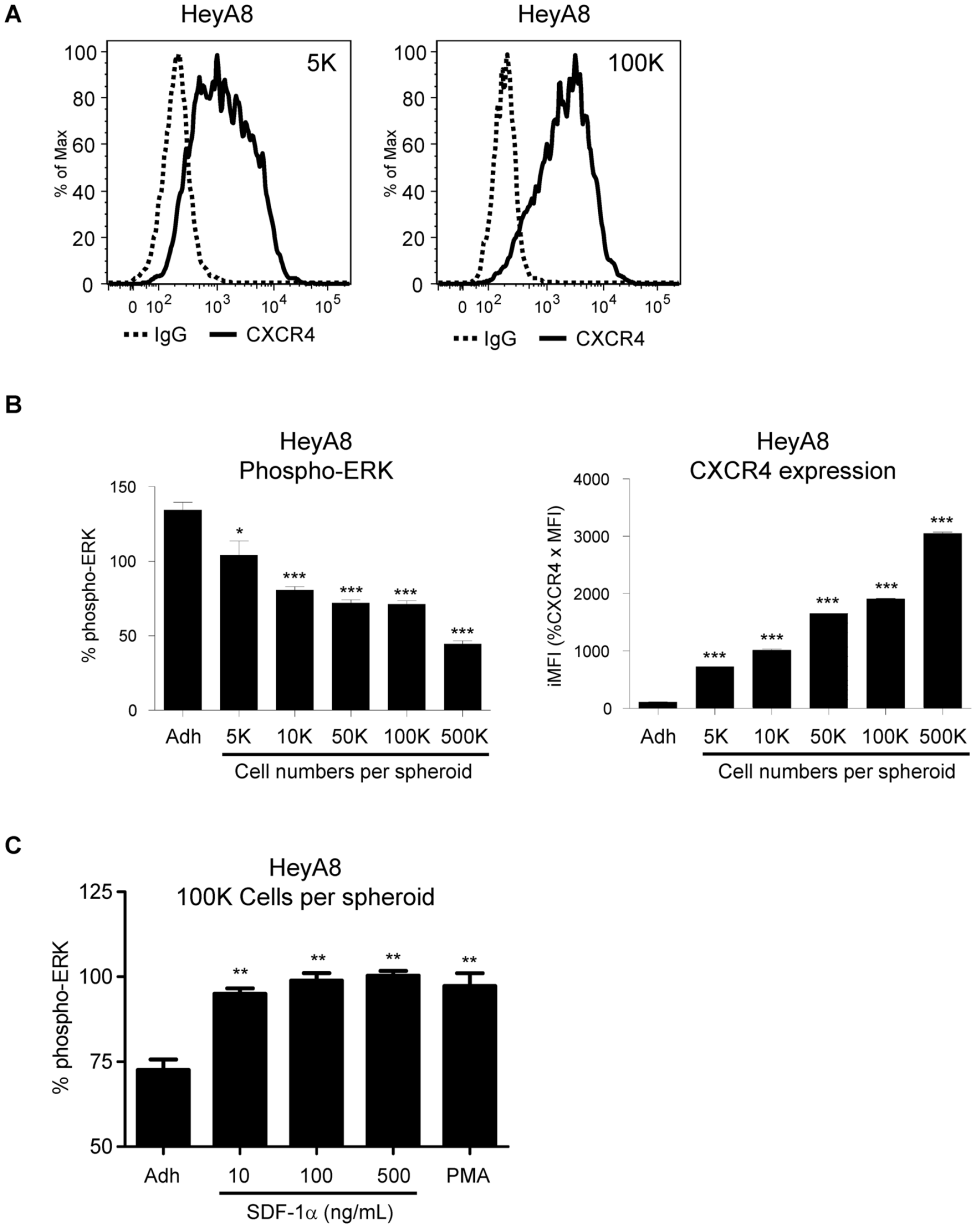
Phospho-ERK level is decreased in 3D spheroids. HeyA8 cells were cultured as 3D spheroids with different number of cells per spheroid. (**A**) At 5000 cells per spheroid, there was a broad shift in CXCR4 surface expression. At higher cell numbers per spheroid, the expression levels of surface CXCR4 increased. (**B**) Corresponding normalized pERK levels showed a strong correlation between increasing CXCR4 surface expression with decreasing phospho-ERK levels. (*, P <0.05 vs Adh, 1-way ANOVA) (***, P <0.0001 vs Adh, 1-way ANOVA). (**C**) At 100,000 cells per spheroid, HeyA8 cells treated with SDF-1α and PMA showed activation of phospho-ERK levels. (**, P <0.005 vs Adh, 1-way ANOVA). Each bar graph is representative of 3 experiments.

Previous result showed that HeyA8 cells have high basal levels of phospho-ERK (Fig. 1B, HeyA8 (−)) when compared to Jurkat T-cells (Fig. 1B, Jurkat (−)) suggest a deregulated ERK pathway in HeyA8 ovarian cancer cell line. It has been reported that aberrant activation of ERK has been linked to BRAF mutations in various cancers including colorectal cancers [30]. We next investigated the correlation between CXCR4 expression and phospho-ERK levels. When HeyA8 cells were cultured with increasing number of cells per spheroid, CXCR4 expression also increased (Fig. 3A, 5K and 100K). Furthermore, the increase in CXCR4 surface expression (Fig. 3B, CXCR4 expression) correlated with decreased phospho-ERK levels (Fig. 3B, Phospho-ERK). To determine if the increased surface CXCR4 was functional, HeyA8 cells cultured as 3D spheroids were treated with SDF-1α. Results showed the activation of ERK pathways as demonstrated by the increase in phospho-ERK levels with both SDF-1α and PMA treatments (Fig. 3C). While every attempt to keep the experiment consistent, we have used different lots of MSD phospho-ERK kit and different passages of the cells which may contribute to differences in the results as shown in Fig. 3C compared to Fig. 3B and 1B in the % phospho-ERK of adherent cells. While variability between assays exists, the experiment in Fig. 3C clearly showed that while using the same cell passage number with that particular MSD phospho-ERK kit, the % phospho-ERK increased with the addition of SDF-1α under 3D spheroid compared to adherent cells. By culturing HeyA8 cells as 3D spheroids, we have demonstrated that the increase in functional CXCR4 expression is correlated with decreasing phospho-ERK levels. Taken together, these results suggest the decrease in phospho-ERK levels correlated with the increase in functional CXCR4 expression. Based on these results, we further investigated the role of ERK signaling pathway in CXCR4 regulation.

**Figure 4:**
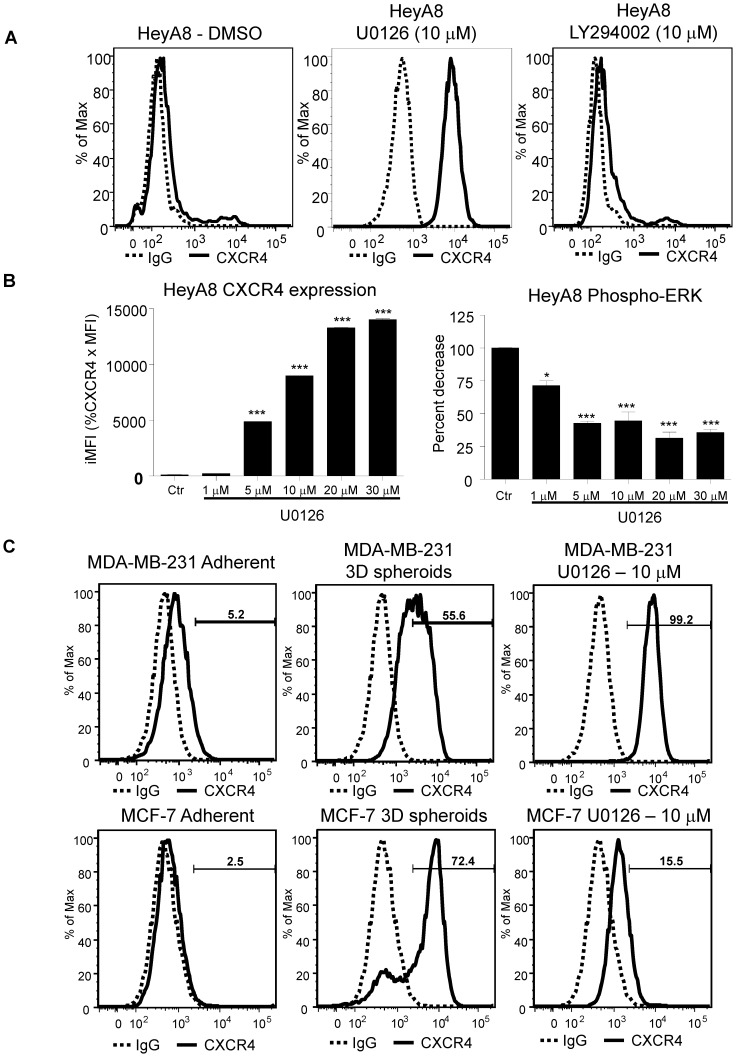
Phospho-ERK inhibition increases CXCR4 surface expression. HeyA8 cells were cultured under normal adherent conditions. 100,000 HeyA8 cells were treated with MEK1/2 inhibitor U0126 and LY294002 at 10 µM for 48 hours. Cells were harvested and analyzed by flow cytometry. (**A**) CXCR4 expression was increased when treated with MEK1/2 inhibitor U0126 as compared to DMSO treated (solid lines). Similar to DMSO treatment, there was no detectable surface CXCR4 expression when treated with PI3K inhibitor LY294002. (*, P <0.05 vs Adh, 1-way ANOVA) (***, P<0.0001 vs Adh, 1-way ANOVA). (**B**) 200,000 HeyA8 cells were plated onto two 6 well plates and treated with U0126. One plate was harvested for CXCR4 expression and the other for phospho-ERK assay. The phospho-ERK assay showed decreasing levels with increasing U0126. Flow cytometry analysis showed a dose dependent increase in CXCR4 expression. (**C**) MDA-MB-231 and MCF-7 cells either grown as 3D spheroids or treated with U0126 showed increase CXCR4 expression similarly to HeyA8 cells. Results are representative of 3 experiments.

**Figure 5:**
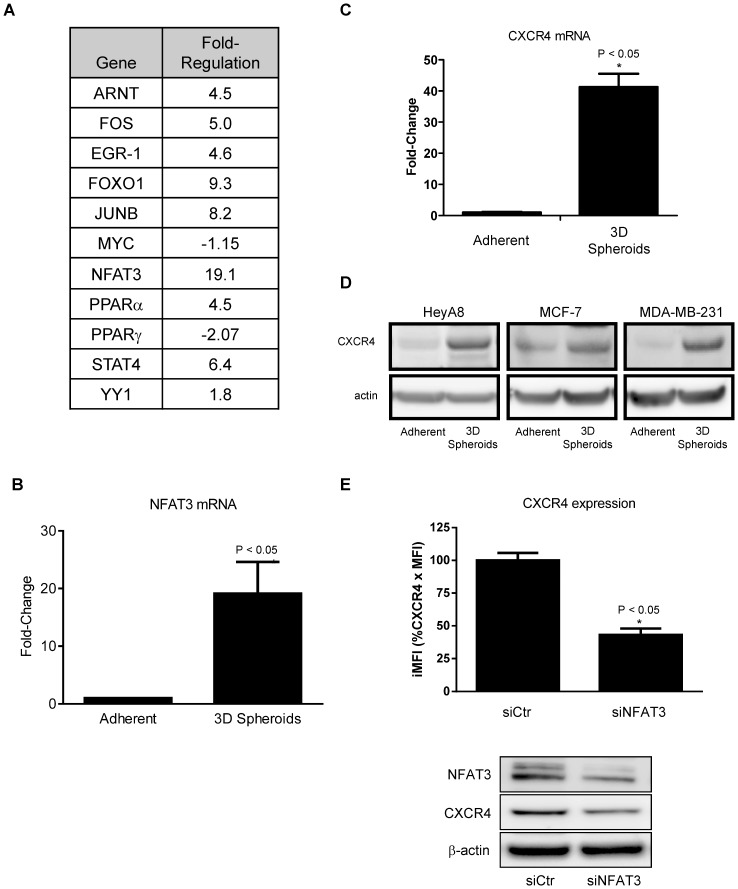
3D spheroids enhance NFAT3 and CXCR4 expression. PCR array of transcription factors was performed according to manufacturer's protocol. The 3D spheroids have 100,000 cells per spheroid. (**A**) A list of transcriptional factors with the corresponding fold change in mRNA expression in cultured 3D spheroids relative to adherent HeyA8 cells. (**B**) NFAT3 mRNA showed an average of ∼ 20 fold increase in the 3D spheroid. (*, P <0.05 vs Adh, 1-way ANOVA). (**C**) Corresponding to the increase in NFAT3 in 3D spheroids, real-time PCR showed a 30-50-fold increase in CXCR4 mRNA in 3D spheroids when compared to adherent HeyA8 cells. (*, P <0.05 vs Adh, 1-way ANOVA). (**D**) Adherent and 3D spheroids western blot analysis of CXCR4 protein in HeyA8, MCF-7, and MDA-MB-231 showed higher CXCR4 levels with 3D spheroids than with adherent cells. (**E**) siRNA targeting NFAT3 decreased the number of CXCR4 positive cells compared to siCtr as determined by flow cytometry. Western blot analysis showed decreased NFAT3 following siNFAT3 vs siCtr and CXCR4 expression also decreased following siNFAT3. Flow cytometry of CXCR4 expression in 3D spheroids were pooled representative cells isolated from at least 10 individual spheroids. Real-time PCR results were normalized to actin. (*, P <0.05 vs siCtr, 1-way ANOVA). The PCR array results are the average of 2 separate experiments, the NFAT3 mRNA, CXCR4 mRNA, siNFAT3, and western blot experiments are representative of 3 separate experiments.

### Phospho-ERK inhibition increases CXCR4 surface expression

In osteosarcoma cells, MEK-ERK and NFkb pathways are involved in SDF-1α mediated cell migration and integrin upregulation [6]. Here we observed a novel relationship between phospho-ERK levels and CXCR4 expression. To further characterize the signaling pathways involved in CXCR4 expression, we used chemical inhibitors of the MEK-ERK and PI3K signaling pathways on adherent HeyA8 cancer cells. Inhibition of MEK-ERK pathways with MEK inhibitor U0126 showed an increase in CXCR4 expression by flow cytometry analysis (Fig. 4A, U0126). However, inhibition of the PI3K pathway with LY294002 did not lead to an increase in CXCR4 expression (Fig. 4A, LY294002). The increase in CXCR4 expression upon U0126 treatment is dose-dependent (Fig. 4B, CXCR4 expression). The phospho-ERK levels also showed a decrease in ERK activity in a dose-dependent manner and it appears that the ERK activity plateau out after 5 µM with no additional decrease (Fig. 4B, Phospho-ERK). These results using chemical inhibitors showed that between MEK-ERK and PI3K pathways, only the MEK-ERK pathway is involved in the increase in CXCR4 expression in HeyA8 ovarian cancer cells. To confirm HeyA8 results, MDA-MB-231 and MCF-7 breast cancer cell lines showed similar increase in CXCR4 surface expression after treatment with MEK inhibitor U0126 and 3D spheroids as compared to adherent (Fig. 4C). Taken together, the levels of phospho-ERK is an important factor in the expression of CXCR4. With the identification of ERK pathway involvement in CXCR4 expression, we next investigated downstream transcriptional factors involved in CXCR4 regulation.

### NFAT3 regulates CXCR4 expression

Previous reports on the regulation of CXCR4 in HIV-1 showed that DNA sequence of -114 to +59 relative to the transcriptional start site in the CXCR4 promoter is critical to the basal transcriptional activity of CXCR4 expression [17], [18]. Gene expression arrays were used to identify transcription factor expression differences between HeyA8 cells grown under 2D adherent and 3D spheroid growth conditions. Transcriptional factors such as ARNT, FOS, EGR-1, FOXO-1, JUN-B, PPARα, YY1, and STAT4 were found to be up-regulated whereas PPARγ and Myc were down-regulated in HeyA8 spheroids compared to the adherent HeyA8 cells (Fig. 5A). Out of 84 transcription factors tested in the gene expression array, the greatest increase came from NFAT3 mRNA with over 20 fold increase in expression on 3D spheroid than 2D adherent cells (Fig. 5B). Furthermore, CXCR4 mRNA was also increased by 30-50 times more in 3D spheroid than 2D adherent cells (Fig. 5C). The increase of CXCR4 mRNA also translates to increase in CXCR4 protein in HeyA8, MCF-7, and MDA-MB-231 cells as shown in the western blot analysis in Fig. 5D. Together, both increases in CXCR4 and NFAT3 mRNA and the corresponding increase in CXCR4 protein suggest a possible connection between NFAT3 transcription factor and CXCR4 expression. To directly test whether NFAT3 plays a role in CXCR4 expression, we used siRNA targeting NFAT3 to determine the effect on CXCR4 expression. Using flow cytometry analysis, knock-down of NFAT3 resulted in a decrease in basal CXCR4 surface expression in HeyA8 adherent cells (Fig. 5E). The NFAT3 knockdown and the decrease in CXCR4 expression were confirmed by western blot analysis showing siNFAT3 lead to a decrease in CXCR4 expression (Fig. 5E).

**Figure 6:**
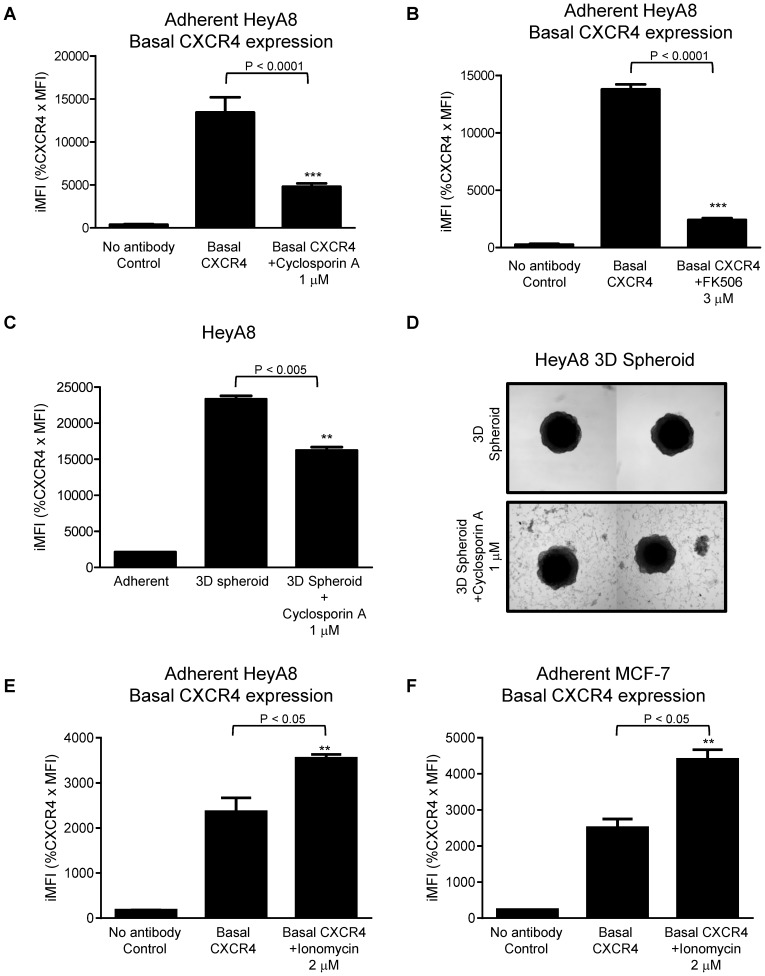
NFAT3 regulates CXCR4 expression. HeyA8 cells were cultured as a monolayer (adherent) and as 3D spheroids as described in materials and methods. HeyA8 adherent cells were treated with calcineurin-NFAT inhibitor cyclosporin A (1 µM) andFK506 (3 µM) for 72 hours. (**A**) Flow cytometry analysis of HeyA8 adherent cells showed basal CXCR4 surface protein expression was attenuated with the treatment of cyclosporin A as compared to no treatment group (Basal CXCR4). (***, P <0.0001 vs. CXCR4, 1-way ANOVA). (**B**) HeyA8 adherent cells treated with FK506 showed basal CXCR4 surface expression decreasing similar to cyclosporine A treated cells. (***, P <0.0001 vs. CXCR4, 1-way ANOVA). (**C**) HeyA8 3D spheroids were treated with Cyclosporin A (1 µM) for 7 days. Flow cytometry showed decreasing levels of surface CXCR4 protein expression treated with cyclosporin A. (**, P <0.05 vs. 3D spheroid, 1-way ANOVA). (**D**) Picture of HeyA8 3D spheroids from Fig. 6C. Cyclosporin A treated 3D spheroids compared to 3D spheroid control showed cells dissociated from the 3D spheroids and attaching to the bottom as adherent monolayer. (**E**)(**F**) Flow cytometry analysis of adherent HeyA8 and MCF-7 cells treated with ionomycin showed significant increase in Basal CXCR4 expression (**, P<0.05 vs. CXCR4, 1-way ANOVA) compared to no treatment (Basal CXCR4). Fig. 6 results are representative of 3 experiments.

Previous figures showed the MEK-ERK pathway is linked to CXCR4 expression, we want to explore further downstream the possible link between NFAT3 pathways in CXCR4 regulation based on the significant increase in NFAT3 mRNA in 3D spheroids by using chemicals to inhibit or activate the NFAT3 pathway. HeyA8 ovarian cells grown under 2D adherent condition were treated with NFAT inhibitor cyclosporin A (CsA), and assessed by flow cytometry (Fig. 6A). As shown under 2D conditions, CXCR4 basal expression is decreased after treatment with cyclosporine A (Fig. 6A, CXCR4 vs. cyclosporine A 1 µM). The decreased basal CXCR4 expression was confirmed with another NFAT inhibitor FK506 (Fig. 6B, CXCR4 vs. FK506 3 µM). To further strengthen the link of NFAT3 on CXCR4 expression, HeyA8 3D spheroids were treated with cyclosporin A. CXCR4 expression when cultured as a 3D spheroid was inhibited by CsA treatment (Fig. 6C, 3D spheroid vs. 3D spheroid + cyclosporine A 1 µM). A picture was taken of the 3D spheroids with and without CsA treatment and compared to control 3D spheroids, CsA treated spheroids showed cells dissociating from the spheroid and appears to grow as a monolayer (Fig. 6D). Up to this point, we have utilized CsA and FK506 to inhibit the NFAT signaling pathway which leads to decrease in CXCR4 expression (Fig. 6A-D). We next determine if CXCR4 expression can be increased by activating NFAT using Ionomycin that increases the intracellular calcium levels leading to NFAT activation. Both HeyA8 and MCF-7 cells treated with Ionomycin showed significant increase in CXCR4 expression thus correlating the results of decreased CXCR4 expression by inhibiting NFAT.

While the knockdown of NFAT3 did not completely abolish CXCR4 expression, these studies targeting NFAT3 through siRNA and chemical inhibitors/activators suggest a link between NFAT transcriptional factor and CXCR4 expression. We next investigated whether activation of NFAT results in binding to the promoter region to drive CXCR4 expression.

### NFAT3 transcriptional activities increase and bind to the CXCR4 promoter

**Figure 7:**
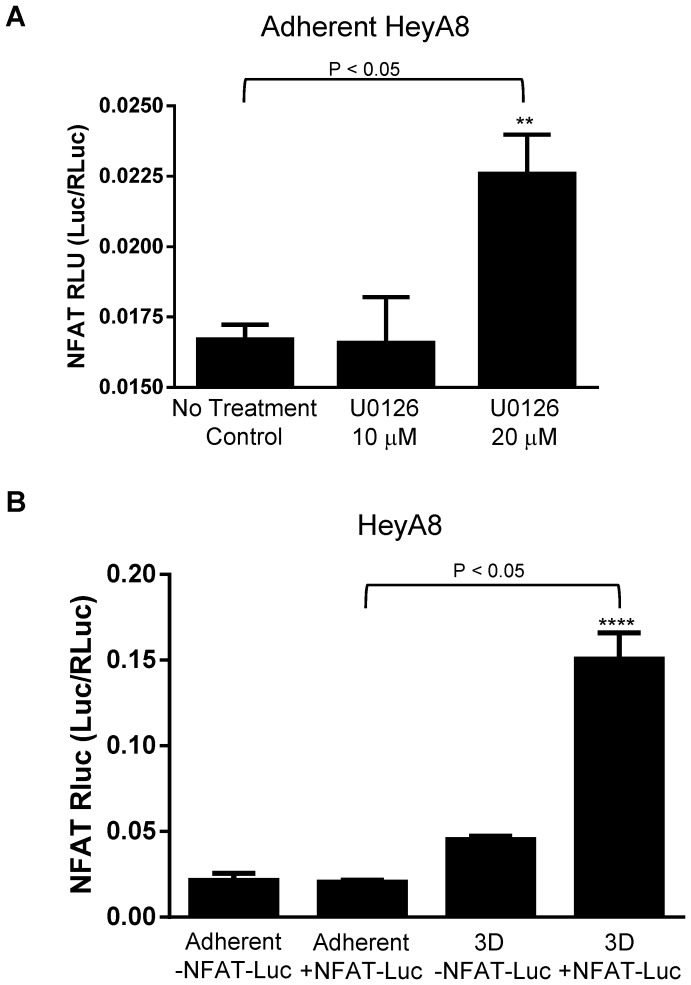
ERK inhibition and 3D spheroids increased NFAT transactivation. HeyA8 cells plated at 50,000 cells in a 24 well plate for transfections. 500 ng of NFAT-Luc/Renilla mixture (40:1) were transfected per well and incubated overnight. U0126 were treated for 16-24 hours and 3D spheroids were formed for 48 hours prior to luciferase assay. (**A**) U0126 treated adherent HeyA8 cells showed slight significant increase of NFAT transactivation at the 20 µm but not at 10 µm. (**B**) 3D spheroid with +NFAT-Luc showed a greater increase in NFAT transactivation compared to adherent HeyA8 cells with +/−NFAT-Luc and 3D spheroid with no NFAT-Luc. (**, P<0.05 vs no treatment control; ****, P<0.05 vs. adherent +NFAT-Luc, 1-way ANOVA). The reporter assays are representative of 3 experiments.

**Figure 8:**
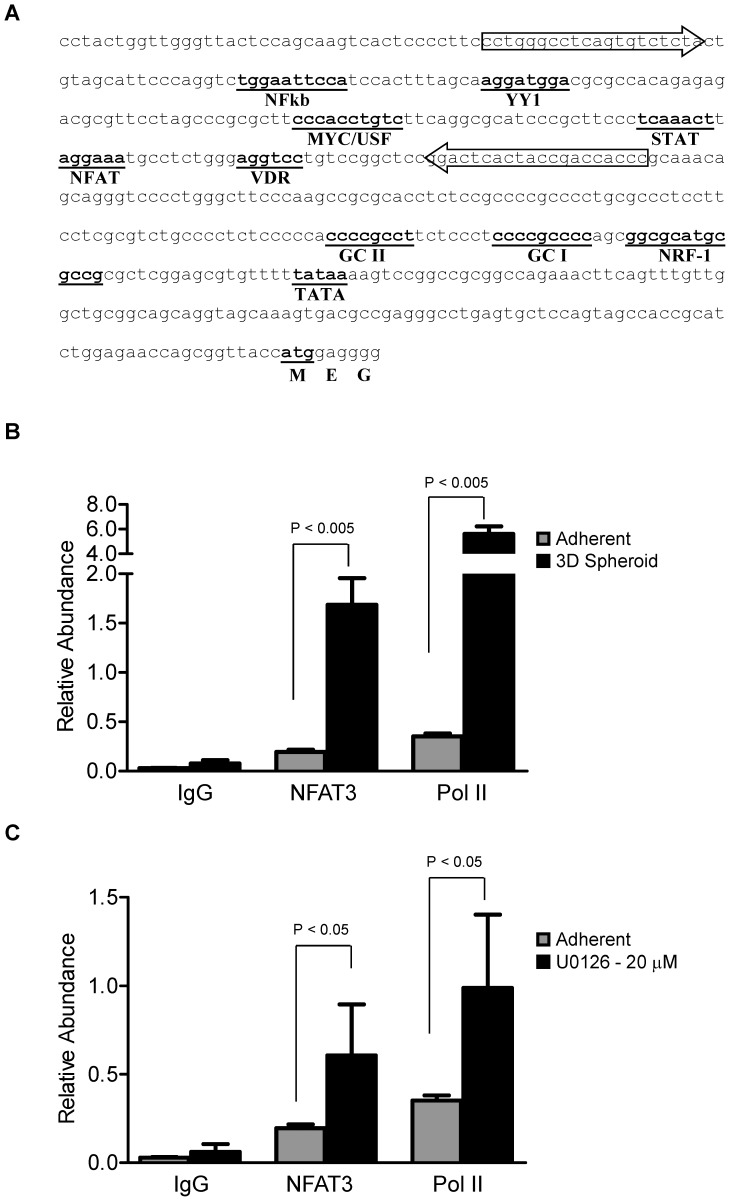
NFAT3 protein binds CXCR4 promoter. HeyA8 cells were cultured as monolayer and as 3D spheroids (100,000 cells per spheroid) as described in materials and methods. (**A**) CXCR4 gene sequence according to GeneBank accession number AF005058. Regions with putative transcriptional factor binding consensus sequences are underlined and in bold. The block arrows indicate real-time PCR primers used for the chromatin immunoprecipitation analysis. (**B**) Chromatin immunoprecipitation of adherent HeyA8 cells and 3D spheroid showed significant (p <0.005) increase in relative abundance of NFAT3 and Pol II on the CXCR4 promoter. (**C**) Similar to 3D spheroids, adherent cells treated with 20 µM of U0126 showed significant (p <0.05) increases in relative abundance of NFAT3 and PolII on the CXCR4 promoter. Average values were derived from 2 independent IPs with qPCRs for each IP performed in duplicate (n = 4).

In HIV-1 entry, c-myc protein enhances while YY1 protein represses CXCR4 promoter activity [31]. In addition to c-myc and YY1, the CXCR4 promoter regions have multiple binding sites for various transcriptional factors including NFkb, c-myc, YY1, STAT, VDR, NRF-1, and NFAT (Fig. 8A) [31] [17], [18]. The NFAT transcriptional binding site on CXCR4 promoter is −260 bp upstream of ATG (Fig. 8A). To determine if the increase in NFAT3 mRNA results in the binding of NFAT3 protein to the NFAT consensus binding motif (T/A)GGAAA, we used the NFAT-Luc reporter assay. HeyA8 cells treated with MEK-ERK inhibitor U0126 showed slight significant increase (∼1.4 fold) in NFAT transactivation at 20 µm but not at 10 µm (Fig. 7A) when compared to no treatment control. When grown as 3D spheroids, NFAT transactivation significantly increased (∼7.5 fold) in 3D spheroids transfected with NFAT-Luc when compared to adherent cells with and without NFAT-Luc and 3D spheroid without NFAT-Luc (Fig. 7B). The NFAT transactivation results corroborated with the increase in NFAT3 mRNA results in Fig. 5B.

To further validate NFAT3 in regulating CXCR4, chromatin immunoprecipitation assay was performed to confirm that NFAT3 does in fact bind to the promoter of CXCR4. Using primers spanning the NFAT binding site (Fig. 8A, arrows), the relative abundance of NFAT3 on the CXCR4 promoter was determined to be significantly higher in 3D spheroids than in adherent cells (Fig. 8B). As expected, the level of Pol II was also significantly higher in 3D spheroids than in adherent cells correlating with an increase in CXCR4 expression. Similar to the 3D spheroid condition, inhibiting the MEK-ERK pathways with U0126 inhibitor also resulted in NFAT3 and Pol II proteins to be significantly higher on the CXCR4 promoter than in adherent cells (Fig. 8C). Taken together, NFAT3 localization and binding to CXCR4 promoter in both conditions (3D spheroid and MEK-ERK inhibition) where high CXCR4 expression was observed suggest a correlation between NFAT3 activation and CXCR4 expression.

## Discussion

In this study, we show that the activation of the ERK pathway inhibits CXCR4 expression. We also demonstrated that under 3D spheroid conditions and ERK inhibition, NFAT3 transactivation increases and binds to the CXCR4 promoter region resulting in an increased CXCR4 expression. These results establish a potential molecular mechanism by which ERK regulates CXCR4 expression through NFAT3 independent of external SDF-1α.

In studying CXCR4 expression of various tumor cell lines, we have observed that suspension tumor cell lines express high levels of CXCR4 while many solid tumors cell lines express low surface CXCR4 by flow cytometry. *In vitro* 3D spheroids have been used previously to study the role of CXCR4 expression in placental vasculogenesis and in the mechanism of human mesenchymal stem cells (hMSCs) adhesion to endothelial cells [25], [32]. Others have used 3D spheroid models to study apoptosis, differentiation, cellular signaling, and cell-cell interactions [33]–[36]. The present study illustrates an *in vitro* method of culturing adherent solid tumor cell lines as 3D spheroids to detect and study CXCR4 expression. We found that a suspension T-cell leukemia cell line Jurkat expresses high levels of SDF-1α responsive CXCR4 receptors while adherent HeyA8 ovarian cell line expresses low levels of CXCR4 and failed to respond to SDF-1α in a phospho-ERK assay (Fig. 1B). Our study also showed that in various adherent cancer cell lines, CXCR4 surface expression increased when grown as 3D spheroids (Fig. 2A–C). It was previously shown that culturing hMSCs as monolayers loses CXCR4 expression which was reversible when grown as 3D spheroids [29]. Therefore, we use the 3D spheroid culturing method to study the mechanisms of CXCR4 regulation in HeyA8 ovarian cancer cell line.

We have also observed that while high CXCR4 Jurkat cells have low basal levels of phospho-ERK, low CXCR4 expressing HeyA8 cells have high levels of phospho-ERK levels (Fig. 1B). It has been reported that constitutive activation of MAP kinase occurs in multiple tumor types, with highest frequencies observed in tumors originating from pancreas, colon, lung, ovary, and kidney while tumors from liver, stomach, esophagus, brain, and hematopoietic cells activate MAP kinase at lower frequencies [37]. In agreement of these findings, we observed high phospho-ERK activity in HeyA8 ovarian cell line and low in Jurkat T-cell leukemia cell line. In respect to high ERK activity in regulating cellular processes, colorectal cancer cells with high ERK activity has been shown to inhibit p27Kip1 cell cycle regulatory protein [38]. In our study, we hypothesized that phospho-ERK activity may contribute to the regulation of CXCR4 expression. To test this hypothesis we cultured HeyA8 cells as 3D spheroids with different cell numbers per spheroid and determined that the increased functional CXCR4 expression correlated with decreased phospho-ERK activity. We showed that by using chemical inhibitor blocking MEK-ERK signaling pathway, we could simulate the enhanced CXCR4 expression seen with the 3D spheroid in HeyA8 cells in adherent culturing conditions (Fig. 4A). However, CXCR4 expression was unchanged when PI3K pathway was blocked suggesting the increase in CXCR4 expression in HeyA8 ovarian cancer cell line is mainly dependent on the MEK-ERK pathway and not the PI3K pathway (Fig. 4A). An interesting observation showed that while there was a dose-dependent increase in CXCR4 expression, phospho-ERK levels seems to plateau off at 10 µM of U0126 with no additional decrease beyond 10 µM. It is possible that with increasing amounts of U0126, additional pathways are being affected. Further investigation is required to determine whether higher concentration of U0126 alters other signaling pathways. We have also confirmed the increased CXCR4 expression with 3D spheroids and ERK inhibition in breast cancer cell lines MDA-MB-231 and MCF7 (Fig. 4C). Reports have shown that SDF-1α/CXCR4 mediated increase in cell migration in lung cancer and osteosarcoma cell lines involve the MEK-ERK-NFkb pathway [6], [39]. While we did not test whether NFkb pathway was involved in CXCR4 expression, it is possible that NFkb and PI3K pathways are more relevant in SDF-1α mediated CXCR4 signaling and not in the basal transcriptional regulation of CXCR4 expression. Together, our findings of increased CXCR4 expression correlating with decreased phospho-ERK levels (3D Sphere condition and small molecule inhibitor of MEK-ERK activity) support the role of phospho-ERK in regulating CXCR4 expression. Since ERK activities are upstream of many transcriptional factors, we sought to determine which downstream factors are involved in regulating CXCR4 expression.

The upstream phosphorylation events during G protein coupled receptor activation lead to the eventual process of desensitization by which the receptors fail to respond further to any additional chemokines [40], [41]. The SDF-1α chemokine ligand has been shown to regulate cell surface CXCR4 expression while IL-2 maintains the basal levels of CXCR4 mRNA [42]–[44]. Earlier work on the CXCR4 promoter showed that NRF-1 and SP1 bind to the proximal region and may regulate the basal level of CXCR4 expression [18]. Further characterization of CXCR4 promoter showed that YY1, a repressor, and c-Myc, an activator, both bind to the promoter region of CXCR4 [31]. YY1 and c-Myc have been shown to interact with each other; over-expression of both proteins in the context of CXCR4 mutually negates their respective functions [30]. In the current study, we screened 84 transcriptional factors from different signaling pathways to determine the role they play in the up-regulation of CXCR4 expression in 3D spheroids. While we did not observe any significant changes in either YY1 or c-Myc expression, which are both known transcriptional regulators of CXCR4, we did see a wide variety of transcriptional factors that were upregulated, in particular NFAT3 which was increased over 20 fold (Fig. 5A–B). The observation that culturing HeyA8 cells as 3D spheroid decreased phospho-ERK activity was intriguing and may be caused by numerous factors. It has been shown that secretion and production of growth regulatory factors are dependent on cell shape, extracellular matrix, or 3D structures [45]. It is plausible that in the 3D spheroid microenvironment, paracrine signaling results in the decrease of phospho-ERK activity that otherwise would not have occurred in normal adherent culturing conditions. While MEK activation and phosphatase activity affect ERK activity, additional investigation on whether HeyA8 cells in 3D spheroid condition alters MEK and phosphatase activity is warranted.

With evidence linking NFAT3 and CXCR4, we further explore the possible role of NFAT3 in regulating CXCR4. We found earlier that the increase in CXCR4 expression when cultured as 3D spheroids can be recapitulated by inhibiting adherent cells with ERK inhibitor. We questioned whether inhibiting NFAT3 pathways in a 3D spheroid model would decrease CXCR4 expression. Indeed, treating HeyA8 3D spheroids with CsA caused a decrease in CXCR4 expression similar to treating adherent HeyA8 cells with CsA (Fig. 6A, 6C). Conversely, activating NFAT activity using Ionomycin increased CXCR4 expression (Fig. 6E–F). The knockdown of NFAT3 using siRNA resulted in lower CXCR4 levels. Together, the results from NFAT inhibition, activation, and knockdown studies confirmed a direct role of NFAT3 in CXCR4 expression (Fig. 5E, 6). While HIF-1α and VEGF regulate CXCR4 expression [46] [47], they themselves are regulated by NFAT. It is possible that knockdown of NFAT3 may affect the regulations of HIF-1α and VEGF thereby affecting CXCR4 expression. Even though CXCR4 expression is decreased with knockdown of NFAT3, further studies looking into NFAT3 regulating CXCR4 expression independent of HIF-1α and VEGF is needed. While knockdown of NFAT3 leads to over 50% decrease in CXCR4 levels, CXCR4 expression was not abolished. This highlights the fact that while NFAT3 signaling pathway can regulate CXCR4 expression, it is likely that other pathways are also involved. Nonetheless, our data illustrates a role for NFAT3 in regulating CXCR4 expression.

The ERK protein is a multi-purpose kinase that has the ability to not only phosphorylate cellular proteins but also translocate to the nucleus and activate transcriptional factors [11]. There have been several reports where ERK is linked to NFAT. In cardiomyocytes, activation of MEK1 increases NFAT4 transcriptional activity in a reporter assay [48]. Furthermore, the same group showed that MEK-1, ERK2, Calcineurin, and NFAT4 all formed a complex to enhance NFAT activation [48]. Another report showed that activation of NFAT activity due to RAS expression or phenylephrine stimulation can be reversed by chemically inhibiting MEK-1 activity [49]. In cardiac myocytes, the activation of RAS-MEK-ERK pathways induces the activation of the NFAT signaling pathway [48], [49]. NFAT is also implicated in T cells where IL-2 mediated cell growth through the T cell antigen receptor involves multiple transcription factors that include NFAT, AP-1, NFkb, and Oct-1 [14], [50], [51]. Although not tested, it is plausible that there is a direct association between ERK and NFAT3 thereby affecting the NFAT3 transcriptional activities. Additionally, MEK activation of ERK may also activate some unknown factors besides calcineurin that directly regulate NFAT3 activity.

A common feature of all the NFAT transcriptional factors is the fact that it is regulated by calcium and calcium/calmodulin-dependent serine phosphatase calcineurin [52]. Normally, activated NFAT in the nucleus is kept there by calcineurin until a decrease in intracellular calcium causes the phosphorylation of NFAT and rapid exit out of the nucleus [13], [52], [53]. In our studies, the increased levels of NFAT3 transcriptional factor suggest a role in regulating CXCR4 expression in the absence of external SDF-1α. Analysis of the CXCR4 promoter region identified a potential NFAT consensus binding sequence (T/A)GGAAA between the STAT and VDR binding sites (Fig. 8A). Furthermore, to determine whether NFAT3 is transcriptionally active, the use of a luciferase reporter under a consensus NFAT binding sequence showed slight significant increase (∼1.4fold) in U0126 treated cells and a greater increase (∼7.5 fold) in 3D spheroid when compared to control (Fig. 7). Finally, with evidence supporting a role for NFAT3 in regulating CXCR4 expression, ChIP analysis of NFAT3 on the CXCR4 promoter showed an enhanced NFAT3 occupancy in both HeyA8 3D spheroid cultures and cells treated with MEK inhibitor. To our knowledge, this is the first report showing that the NFAT3 transcriptional factor directly binds to the promoter of CXCR4 and this binding may potentially increase CXCR4 expression. Future work looking at NFAT3 transactivation activity on CXCR4 promoter using point mutations will directly link the importance of NFAT3 in regulating CXCR4 expression. In T-cells, PMA-induced up-regulation in CXCR4 mRNA was inhibited by the addition of calcium ionophore ionomycin and this inhibition was reversed with Cyclosporin A (calcineurin-NFAT inhibitor) [12]. The results from Cristillo and Bierer 2003 [12] are in contradiction to our studies since there were no changes in CXCR4 mRNA levels between PMA treatment alone and PMA plus CsA co-treatment. One obvious difference between our work and Cristillo and Bierer is the fact that we used adherent cancer cell line and Cristillo and Bierer used human peripheral blood lymphocytes. Even in our studies, we saw differences between Jurkat T-cell leukemia and HeyA8 ovarian cancer cell lines with regards to phospho-ERK levels and CXCR4 surface expression levels, further illustrating the stark differences in regulation and signaling between cell types. Typically, an increase in intracellular calcium triggers a wide variety of cellular functions involved in cell growth, death, and differentiation [54]–[56] However, a report by Dolmetsch et al. 1997 [57] showed that in B-lymphocytes, NFkb and JNK signaling pathways were activated by a large transient rise in calcium levels whereas the NFAT signaling pathway was activated by a low sustained calcium levels. In our studies, we have observed lower calcium levels in both 3D spheroids and MEK-ERK inhibition (data not shown) which contradicts conventional thinking of increased calcium levels in the activation of NFAT activity. However, when treating adherent HeyA8 cells with Ionomycin to increase intracellular calcium levels, we detected an increase in surface CXCR4 levels (Fig. 7). Therefore, further investigation is required to determine whether the observed decrease in calcium levels is the result of increased CXCR4 expression or a response to ERK inhibition to regulate NFAT3 signaling pathway. With NFAT3 activation and regulation of CXCR4 expression, there exists the possibility of additional factors that may stabilize NFAT3 independent of calcium/calcineurin activity. In support of this possibility, Kuroda et al. 2012 [58] showed that Cot, a serine/threonine kinase directly phosphorylates and stabilizes all calcium/calcineurin regulated NFAT transcriptional factors during osteoclastogenesis.

In this paper, we have utilized a 3D spheroid culturing condition to study the regulation of CXCR4 in solid tumor cell lines. Our studies showed that ERK plays a role in regulating CXCR4 expression through the NFAT signaling pathways. We observed in a NFAT reporter assay that while 3D spheroids condition caused a ∼7.5 fold increase in NFAT transactivation activity compared to adherent, there was only ∼1.4 fold increase with U0126 treated cells. It appears that in 3D spheroid culturing conditions, NFAT3 transcription factor may play a dominant role in regulating CXCR4 expression but not in the case of U0126 treated cells. We further showed for the first time that NFAT3 transcriptional factor localizes and binds to the promoter of CXCR4 in HeyA8 ovarian cell line in a ChIP assay. However, this does not negate other transcriptional factors that have binding sites on the CXCR4 promoter in either positively or negatively regulating CXCR4 expression. The U0126 treated ChIP result showed less NFAT3 binding to the promoter when compared to the 3D spheroids which may explain why in the previous NFAT reporter assay that 3D spheroids showed a greater fold increase in NFAT transactivation than U0126 treated cells. We have also observed in a reporter assay that when HeyA8 cells were treated with MEK inhibitor, NFkb and STAT transcriptional factors were up-regulated whereas Myc and YY1 were down-regulated (data not shown). Further investigations are required to determine whether these transcriptional factors act synergistically with or independently from NFAT3 to regulate CXCR4 expression and what additional factors contribute to CXCR4 expression during U0126 inhibition of phospho-ERK.

**Figure 9:**
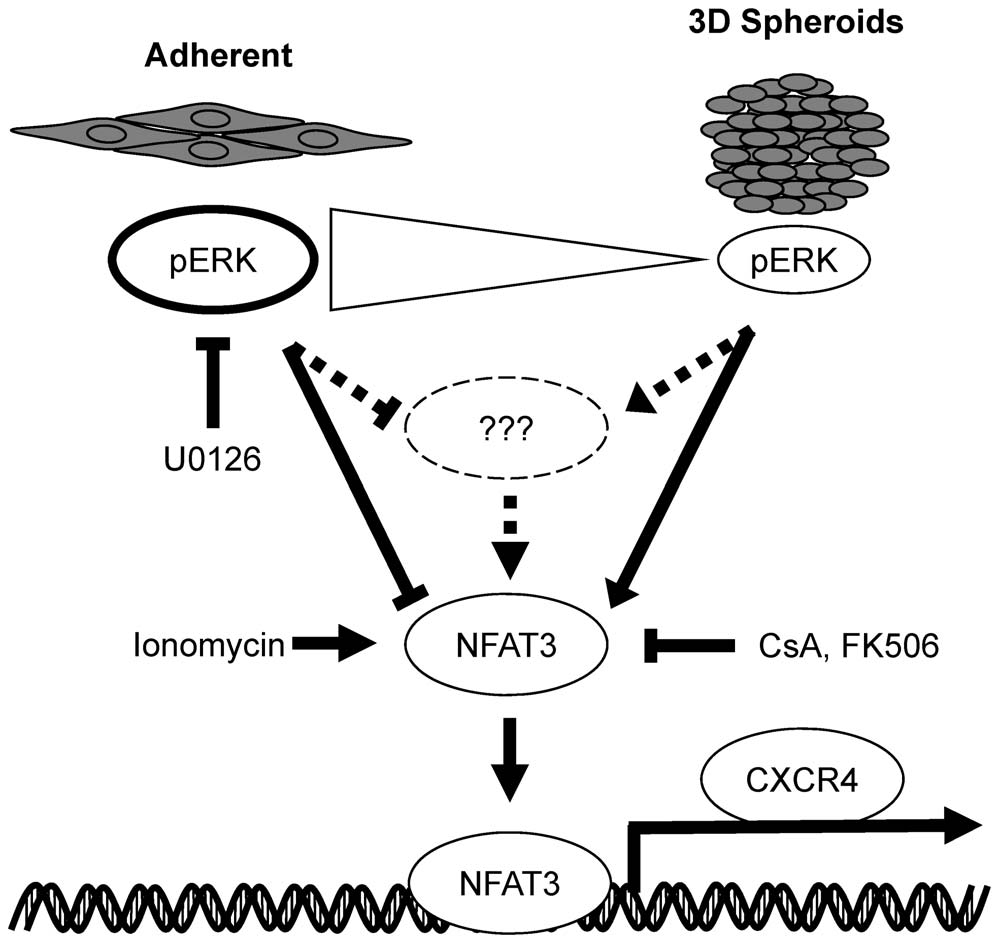
Model for ERK-NFAT mediated regulation of CXCR4 expression. Our findings suggest that the enhanced phospho-ERK levels in adherent cells prevent NFAT3 mediated CXCR4 up-regulation. The increase in phospho-ERK levels may directly or indirectly inhibit NFAT3 activity but when phospho-ERK levels were decreased through 3D spheroid culturing or treatment with MEK inhibitor U0126, activated NFAT3 transcriptional factor localized to the nucleus and occupied the CXCR4 promoter to activate CXCR4 expression.

CXCR4 has been shown to be one of the key drivers in tumor progression as we have seen that knockdown of CXCR4 in a HeyA8 xenograft model showed tumor growth inhibition (data not shown). The findings from this study showed for the first time that the ERK-NFAT3 signaling pathways are linked to the regulation of CXCR4 expression. Based on the results presented here, we propose a model where increased activation of phospho-ERK negatively regulates CXCR4 expression either indirectly by an unknown factor or directly through the NFAT3 transcription factor (Fig. 9). The Model showed that when grown under 3D spheroid conditions, phospho-ERK levels decreased when compared to adherent conditions (Fig. 3B, phospho-ERK) which also resulted in an increase in basal CXCR4 expression (Fig. 2; Fig. 3B, CXCR4 expression). With higher phospho-ERK levels in adherent cells, inhibition of ERK pathway with U0126 in adherent growth conditions showed similar increase in CXCR4 expression as the 3D spheroids suggesting a correlation between phospho-ERK levels and CXCR4 expression (Fig. 4). We further identified NFAT3 transcriptional factor as a key component in regulating basal CXCR4 expression when grown in 3D spheroids (Fig. 5A, B). Further analysis using activator and inhibitors of the NFAT pathway (Fig. 6) showed NFAT3 regulating CXCR4 expression and was further confirmed showing NFAT3 binding to the CXCR4 promoter (Fig. 8). While additional studies are required to provide a direct link between phospho-ERK and NFAT3, our results using 3D spheroids and pathway inhibitors have revealed a novel role for ERK and NFAT3 in regulating CXCR4 expression. These discovered findings here illustrate additional pathways that regulate CXCR4 and provide an understanding how conventional chemotherapeutic drugs targeting MEK-ERK and NFAT pathways change target expression. Since we provide evidence of how chemotherapeutic drugs may alter CXCR4 expression, it may be an attractive approach to combine conventional therapy with antibody-based targeted therapies for cancer.
